# Primary health care utilization for alcohol-attributed diseases in British Columbia Canada 2001–2011

**DOI:** 10.1186/s12875-015-0247-4

**Published:** 2015-03-11

**Authors:** Amanda K Slaunwhite, Scott Macdonald

**Affiliations:** Department of Sociology, University of New Brunswick and Centre for Addictions Research of British Columbia, PO Box 1700 STN CSC, Victoria, BC V8W 2Y2 Canada; Centre for Addictions Research of British Columbia and School of Health Information Science, University of Victoria, 2300 McKenzie Ave, Rm. 281, Victoria, BC V8P 5C2 Canada

## Abstract

**Background:**

The purpose of this study was to determine whether general practitioner visits for alcohol-attributed diseases increased in a decade when several regulatory changes were made to the distribution and price of alcohol in British Columbia Canada.

**Methods:**

General practitioner consultations for alcohol-attributed diseases were examined using data from British Columbia’s Medical Services Plan database. Negative binomial regression was used to measure the significance of yearly variations using incidence rate ratios by disease type per year.

**Results:**

From 2001 to 2011, 690,401 visits were made to general practitioners by 198,623 persons with alcohol-attributed diseases. Most visits (86.2%) were for alcohol dependency syndrome (N = 595,371). General practitioner visits for alcohol-attributed diseases increased significantly (p < .001) by 53.3% from 14,882 cases in 2001 to 22,823 cases in 2011. While the number of cases increased from 2001–2011, the frequency of visits to general practitioners significantly decreased from 3.9 in 2001 to 2.7 visits per case in 2011 (F = 428.1, p < .001).

**Conclusion:**

From 2001 to 2011 there were significant increases in the number of persons presenting to general practitioners with alcohol-attributed diseases in British Columbia. The results of this study demonstrate the need to provide enhanced support to general practitioners in the treatment of patients with substance use disorders given the increasing number of primary health care patients with alcohol-attributed diseases.

## Background

In the past decade there has been a renewed focus on leveraging opportunities in primary health care to reduce health inequities through regular screening and health promotion counseling that work to detect illnesses early in their development and address negative health behaviors among patient populations [1-3]. The importance of primary health care to the identification of persons at risk of developing subsequent mental and physical health conditions is highly apparent in relation to alcohol consumption, which is a significant contributor to premature mortality in Canada [4,5]. Previous research on health care use and alcohol consumption in British Columbia (BC) has focused almost exclusively on secondary and tertiary level substance use treatment services that are accessed by only a small proportion of all at-risk drinkers in the province [6]. The purpose of this project was to address this knowledge gap by determining if there were increases in general practitioner (GP) visits for alcohol-attributed diseases (AADs) from 2001 to 2011.

GPs are the most accessible health service available to persons with high levels of alcohol consumption in Canada [7]. GP billing for treatment of AADs is a strong measure of disease symptomology and potential service need among the population because GPs are the most accessible health service in both urban and rural areas. GPs are gatekeepers to secondary or tertiary services that require physician referral, and they are in an optimal position to deliver effective brief interventions to reduce alcohol consumption [8-10]. Research has found that drinkers are much more likely to discuss problems related to alcohol consumption with their regular family doctor than any other type of health care provider because of their doctor’s existing rapport and historical knowledge of the patient [11-13].

Data from 2001–2011 were used to study trends in primary health care use for AADs. During this period there were substantial increases in per capita consumption of alcohol, and several regulatory changes introduced that led to the opening of private liquor stores throughout the province, and incremental increases to the minimum price of alcohol products [14].

## Methods

### Measures

A count of ‘cases’ by disease type and year refers to the number of unique individuals presenting with an AAD to a GP in any given year (January 1, 2001 to December 31, 2011) whereas the count of ‘visits’ refers to all unique encounters to a GP by persons with AADs. The age and sex of patients were derived from the MSP Registry Demographics Collection. The service location of each billing record was grouped into 4 main categories: GP offices in the community; emergency rooms (ERs); hospitals, and all other locations. The AADs examined in this paper are described by their ICD-9 code in Table 1. The diseases selected for this project are wholly attributed to alcohol consumption: alcoholic-related psychoses (291, 291.0-291.8); alcohol dependence syndrome (303.0); alcohol abuse (305.0); alcoholic polyneuropathy (357.7); alcoholic cardiomyopathy (425.5); alcoholic gastritis (535.3); alcoholic fatty liver (571.0); acute alcoholic hepatitis (571.1); alcoholic cirrhosis of the liver (571.2), and unspecified alcohol-related liver damage (571.3).

**Table 1:** **Alcohol-attributed disease by ICD 9 code**

**ICD-9 code** ^**a**^	**Disease type**
291, 291.0-291.8	Alcoholic psychoses; Delirium tremens; Korsakov psychosis; Other alcoholic dementia; Alcoholic hallucinosis; Pathological drunkenness; Alcoholic jealousy; Other alcohol psychosis; Unspecified alcohol psychosis
303.0	Alcohol dependence syndrome
305.0	Alcohol abuse
357.7	Alcoholic polyneuropathy
425.5	Alcoholic cardiomyopathy
535.3	Alcoholic gastritis
571.0	Alcoholic fatty liver
571.1	Acute alcoholic hepatitis
571.2	Alcoholic cirrhosis of the liver
571.3	Alcoholic liver damage, Unspecified

^a^ICD-9 refers to the *International Classification of Diseases, Ninth Revision.*

### Data source

Physician billing data was used to measure changes in primary health care utilization for AADs from January 1, 2001 to December 31, 2011. The BC Ministry of Health approved access to, and use of, Medical Services data via Population Data BC for this study [15-17]. This project was also approved by the University of Victoria Human Research Ethics Board (Protocol Number: 13–454).

**Figure 1:**
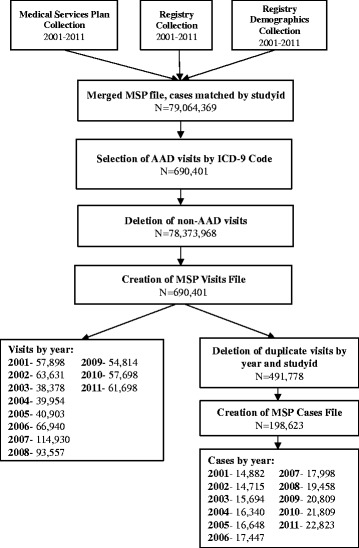
**Database creation flow chart.**

The Medical Services Plan (MSP) File contains data on all claims made by fee-for-service practitioners for patients covered by BC’s universal health insurance program since 1985. All claims made by fee-for-service practitioners for persons covered by BC’s universal health insurance program are included in the MSP file and each claim is coded with an *International Classification of Diseases – Ninth Revision* (ICD-9) code. The Registry Demographics Collection data file contained patient information such as the date of birth and sex. The Registry Collection database contained geographic information about patients, such as the location of their mailing address by Health Service Delivery Area (HSDA). The ‘visits’ and ‘cases’ databases were created using the schematic outlined in Figure 1. Cases from these databases were matched by patient’s unique study identification numbers. The data was linked by AS and extracted three times to minimize data errors. SM reviewed the results to identify any potential coding errors.

### Descriptive methods

Descriptive statistics were calculated using the frequency, means-test, and cross-tabs functions in SPSS 22. The cases per 100,000 persons were calculated using population data by year for the Province of BC from BC Stats [18]. ANOVA tests were used to measure the significance of year-to-year differences for each AAD, and the Durbin-Watson statistic was used to determine if serial autocorrelation was present in the data, and the independence of cases by year. Cells with less than 30 cases were suppressed, and to address small sample sizes, the cases and visits for alcoholic polyneuropathy, cardiomyopathy, and gastritis were grouped for data analysis to study trends by year.

### Time series methods

Negative binomial regression was used to measure the significance of yearly variations in the number of persons presenting with AADs. Results of the initial Poisson regression model showed that the data was overdispersed, as demonstrated in high chi-square values that were statistically significant (p < .05). Because of this overdispersion, negative-binomial regression was used to model the counts of persons per year by AAD to derive the exponentiated coefficients (exp(*β*)values) for each year by AAD [19,20]. These values are interpreted as IRRs because they measure changes to the count of cases in comparison to the reference year (2001) count of cases [21-23].

## Results

### Demographics

From 2001–2011, 66.2% (N = 131,454) of all persons that saw a GP for AADs were male. For all disease types, there were more males than females that saw a GP for treatment of AADs, however there was some variation as described in Table 2. Males represented 70.8% of all cases of acute alcoholic hepatitis, but only 56.3% of all alcoholic fatty liver cases. Persons included in this study were on average 45.9 years of age, however there was some variation by AAD type. Persons presenting with alcohol abuse were the youngest with a mean age of 41.3 years compared to persons with liver cirrhosis that were the eldest at 58.8 years of age.

**Table 2:** **Sample characteristics by alcohol-attributed disease type (2001–2011)**

**ICD-9 definition**	**ICD-9 code**	**% Visits (n)** ^**a**^	**% Cases (n)** ^**b**^	**Age**	**Gender**
				$\overline{x}$	**% Male (n)**
Alcoholic psychoses	**291**	3.8 (26,400)	3.6 (7,094)	57.9	60.8 (4,313)
Alcohol dependence syndrome	**303**	86.2 (595,371)	84.1 (167,057)	45.6	66.9 (111,753)
Alcohol abuse	**305.0**	5.8 (39,983)	7.8 (15,502)	41.3	61.8 (9,586)
Alcoholic polyneuropathy, cardiomypathy, gastritis	**357.7, 425.5, 535.3**	.8 (5,257)	.9 (1,858)	43.7	67.6 (1,256)
Alcoholic fatty liver	**571.0**	1.0 (6,960)	1.6 (3,109)	51.5	57.3 (1,781)
Acute alcoholic hepatitis	**571.1**	.6 (4,329)	.7 (1,316)	50.9	70.8 (932)
Alcoholic cirrhosis liver	**571.2**	1.4 (9,960)	1.1 (2,106)	58.8	68.1 (1,434)
Alcoholic liver damage unspecified	**571.3**	.3 (2,141)	.3 (581)	54.3	68.7 (399)
**Total or** $\overline{x}$ **(n)**	**-**	**100 (690,401)**	**100 (198,623)**	**45.9**	**66.2 (131,454)**

^a^Cases represent unique individuals.

^b^Visits refer to all unique health care encounters.

### Service characteristics

In the 10-year period there were 4,657.6 AAD cases per 100,000 persons in BC. The mean number of visits per case varied by disease type, with an average of 3 GP visits per person from 2001–2011. Patients with alcoholic fatty liver had the lowest number of visits per case (1.7 visits) compared to patients with alcoholic cirrhosis of the liver that had an average of 4.7 GP visits per case. The service locations of GP visits are described in Table 3. There were 690,401 visits to GPs for AADs from 2001 to 2011, and 65.9% of these visits took place in GP offices in the community.

**Table 3:** **New and repeat cases by year (2001–2011)**

**Year**	**Repeat years range**	**Repeat reference years**	**Repeat % (n)**	**New cases % (n)**
2002	2001	1	25.3% (3,727)	74.7% (10,988)
2003	2001-2002	2	32.0% (5,026)	68% (10,668)
2004	2001-2003	3	37.1% (6,054)	62.9% (10,286)
2005	2001-2004	4	39.9% (6,650)	60.1% (9,998)
2006	2001-2005	5	41.8% (7,299)	58.2% (10,148)
2007	2001-2006	6	43.7% (7,864)	56.3% (10,134)
2008	2001-2007	7	44.0% (8,565)	56.0% (10,893)
2009	2001-2008	8	46.0% (9,581)	54.0% (11,228)
2010	2001-2009	9	47.6% (10,388)	52.4% (11,421)
2011	2001-2010	10	49.2% (11,233)	50.8% (11,590)

### Time series trends: cases

**Figure 2:**
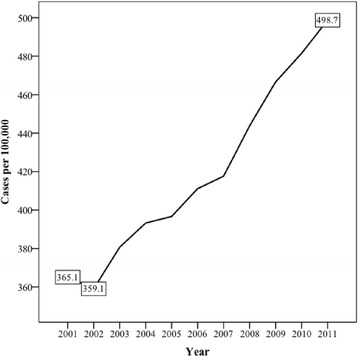
**Cases per 100,000 by year for all alcohol-attributed diseases (2001–2011).**

There was a 53.3% increase in the total number of persons presenting to GPs with AADs from 14,882 cases in 2001 (365.1 per 100,000) to 22,823 cases (498.7 per 100,000) in 2011 (Figure 2). This growth was largely attributed to GP visits by new cases as opposed to repeated health care use by persons that had previously seen a GP for an AAD (Table 4). The greatest increases in cases were attributed to alcohol abuse, alcoholic fatty liver, and alcoholic cirrhosis of the liver. Table 4 contains the number of cases per 100,000 persons by AAD and year, and Table 5 describes the corresponding IRR values and ANOVA (F) results.

**Table 4:** **Cases per 100,000 persons by alcohol-attributed disease and year (2001–2011)**

	**Alcohol psychoses**	**Alcohol dependence syndrome**	**Alcohol abuse**	**Alcoholic polyneuropathy, cardiomypathy, gastritis**	**Alcoholic fatty liver**	**Acute alcoholic hepatitis**	**Alcoholic cirrhosis liver**	**Alcoholic liver damage unspecified**	**All AADS**
2001	10.5	337.2	7.1	2.2	3.9	1.9	1.6	+^a^	365.0
2002	11.2	328.1	9.7	1.9	4.5	1.1	1.8	+	358.9
2003	10.6	343.6	14.6	2.5	4.9	1.4	2.4	+	380.6
2004	13.5	347.4	19.2	3.4	4.5	1.9	2.5	0.8	393.3
2005	13.1	342.9	25.4	3.7	4.6	2.6	3.5	0.9	396.8
2006	14.4	349.9	27.8	5.2	5.5	2.7	4.2	1.4	411.3
2007	13.9	355.6	29.8	5.0	5.8	3.1	4.5	1.6	419.4
2008	18.3	371.4	36.8	4.9	6.4	3.1	4.9	1.4	447.4
2009	19.5	389.0	39.8	4.0	7.7	3.7	6.3	1.8	471.8
2010	20.0	375.3	64.5	4.9	9.6	4.0	7.9	2.1	488.3
2011	19.9	370.9	81.2	5.4	14.4	4.8	8.9	1.7	507.3
$\overline{x}$	**15.0**	**355.6**	**32.4**	**3.9**	**6.5**	**2.8**	**4.4**	**1.5**	**421.8**

^a^Suppressed data (N = <30).

**Table 5:** **Incidence rate ratios (exp (** ***β*** **)) for cases by alcohol-attributed disease and year (2001–2011)**

	**Alcohol psychoses**	**Alcohol dependence syndrome**	**Alcohol abuse**	**Alcoholic polyneuropathy, cardiomypathy, gastritis**	**Alcoholic fatty liver**	**Acute alcoholic hepatitis**	**Alcoholic cirrhosis liver**	**Alcoholic liver damage unspecified**
**2002** ^**a**^	1.1	1.1**	1.4***	0.9	1.2	.6**	1.1	+^c^
**2003**	0.9	1.3***	1.9***	1.1	1.2	0.7	1.4*	+
**2004**	1.2**	1.5***	2.5***	1.4*	1.1	0.9	1.5*	+
**2005**	1.1*	1.8***	3.3***	1.5**	1.1	1.2	2.1***	1.3
**2006**	1.2**	1.9***	3.5***	2.1***	1.3*	1.3	2.4***	1.9**
**2007**	1.2*	1.9***	3.7***	1.9***	1.3*	1.34*	2.5***	2.2**
**2008**	1.4***	2.2999	4.3***	1.8***	1.3**	1.3*	2.6***	1.7*
**2009**	1.4***	2.3***	4.4***	1.4*	1.5***	1.5**	3.1***	2.1*
**2010**	1.4***	3.0***	6.8***	1.6***	1.8***	1.6**	3.7***	2.3***
**2011**	1.4***	3.5***	8.3***	1.7***	2.6***	1.8**	4.1***	1.7**
**DW** ^**b**^	1.8	1.7	1.8	1.8	1.6	1.8	1.5	1.8
**F (sig)**	101.2***	4770.6***	4441.9***	44.9***	230.4***	87.8***	333.4***	49.6***

^a^Reference year 2001.

^b^Durbin Watson test statistic.

^c^Suppressed data (N = <30).

*p < .05; **p < .01; ***p < .001.

There was a significant increase in the number of persons per 100,000 seeing a GP for alcohol abuse (ICD-9 305.0) from 7.1 persons per 100,000 in 2001 to 81.2 persons per 100,000 in 2011. In the 10-year period, the second largest growth in cases was for alcoholic cirrhosis of the liver that increased from 1.6 cases per 100,000 in 2001 to 8.9 cases per 100,000 in 2011. The third largest increase in AAD cases was for alcoholic fatty liver disease. There was a 368% increase in the number of persons presenting with alcoholic fatty liver disease from 3.9 persons in 2001 to 14.4 persons per 100,000 in 2011. This growth largely occurred from 2007 onward, resulting in significant IRR increases in the 2007–2011 period.

### Time series trends: service frequency

Although the number of cases of AADs incrementally increased since 2001, different trends were observed in the frequency of visits over the 10-year period. The average number of visits per case by AAD and year are described in Table 6. ANOVA tests for year to year differences per AAD were significant (p < .001) for all disease groups. From 2001 to 2007 there were steady increases in the average number of GP visits per AAD. The largest increases in the average number of visits per case from 2001 to 2007 were for alcohol psychoses (64% increase); alcoholic polyneuropathy, cardiomyopathy, and gastritis (90% increase), and acute alcoholic hepatitis (75.8% increase). The average number of visits per AAD peaked in 2007 and subsequently declined for most diseases from 2008 through 2011. These decreases in the average number of visits to a GP by persons with AADs have fallen below 2001 levels of utilization for most AAD types.

**Table 6:** **Average frequency of visits by alcohol-attributed disease and year (2001-2011)**

	**Alcoholic psychoses**	**Alcohol dependence syndrome**	**Alcohol abuse**	**Alcoholic polyneuropathy, cardiomypathy, gastritis**	**Alcoholic fatty liver**	**Acute alcoholic hepatitis**	**Alcoholic cirrhosis liver**	**Alcoholic liver damage unspecified**	**All AADs**
**2001**	3.7	3.4	3.1	2.6	2.9	3.2	5.2	+^a^	3.9
**2002**	3.8	3.6	3.3	3.5	3.1	3.4	4.9	+	4.3
**2003**	2.5	2.4	2.1	2.2	1.8	2.4	4.6	+	2.4
**2004**	2.7	2.4	2.2	1.9	1.7	3.1	4.8	2.0	2.4
**2005**	2.8	2.6	2.4	2.5	1.8	3.0	4.1	2.9	2.5
**2006**	3.7	3.6	3.7	3.3	2.6	3.4	5.2	2.9	3.8
**2007**	6.1	5.4	5.0	4.9	4.6	5.7	7.9	5.6	6.4
**2008**	4.3	4.0	3.6	3.5	3.8	4.6	6.3	4.1	4.8
**2009**	3.3	2.6	2.1	2.1	1.5	2.3	4.3	4.2	2.6
**2010**	3.5	2.5	2.1	2.9	1.4	2.3	4.4	2.3	2.6
**2011**	3.8	2.6	2.4	2.3	1.4	2.6	4.1	2.9	2.7
$\overline{x}$ **(2001-2011)**	3.7	3.1	2.7	2.9	2.2	3.2	5.0	3.5	3.5
**F (sig)**	71.2***	112.4***	72.6***	10.9***	46.6***	8.5***	5.2***	2.3*	428.1***

^a^Suppressed data (N=<30).

*p<.05 ***p<.001.

## Discussion

In this study, the average age of persons that saw a GP for AADs was 45.9 years, with some variation by AAD type, which is similar to the age range of alcohol-attributed mortality cases in BC [4,24]. Over 66% of persons that saw a GP from 2001–2011 for AADs were male, which is consistent with previous studies that have found that men are more likely to become heavy drinkers and develop AADs compared to females [25]. Persons with alcohol abuse tended to be younger than persons with other AADs, whereas persons with liver cirrhosis had the eldest average age of 58.8 years. Alcohol abuse is generally more common among younger male drinkers, and is typically associated with experiencing the acute harms of high-risk alcohol consumption without dependency, such as injuries due to hazardous behaviors while intoxicated [26]. In comparison, alcohol dependency is more common among persons over age 40 and persons with alcohol dependency experience chronic physical health issues as the result of alcohol consumption, including withdrawal symptoms and liver damage [10,27,28]. Previous research has found that many younger drinkers with alcohol abuse under 40 years of age do not develop many of the chronic AADs described in this paper, such as alcoholic psychoses and alcoholic liver cirrhosis [26,29]. In comparison, persons with alcohol dependency are usually older (40–50 years of age) and experience other physical health issues as the result of chronic, long-term alcohol consumption such as liver or neurological brain damage [30,31].

Over 86% of GP visits in this study were for alcohol dependency syndrome. However, in comparison, there were only 15.6 hospital discharges per 100,000 for alcohol dependency syndrome in 2011, compared to 56.5 discharges for alcoholic psychoses [32]. This suggests that GPs are more widely accessed by persons with alcohol-dependency issues in BC compared to other health service types.

In this study, 65.9% of all visits occurred in family doctors’ offices in the community with some variation by AAD type. Cases of alcoholic psychoses comprised a significant proportion of all GP consultations in ERs and this is echoed in hospital discharge data from 2011 [32]. Increased use of hospital and ERs by persons with alcoholic psychoses could be associated with the intensity of treatment required, particularly for management of withdrawal symptoms, and comorbid mental health and substance use dependency issues [10]. There was also greater use of ERs by persons with alcohol abuse in this study compared to all other AADs. This could be associated with the presentation of alcohol related injuries more often in ERs (e.g. motor vehicle accidents) than family doctors’ offices [33].

### Time series trends: cases

There was a 53.3% increase in the number of persons seeking treatment for an AAD from GPs in BC from 14,882 cases in 2001 to 22,823 cases in 2011. The increasing number of cases is attributed predominantly to ‘new cases’ or persons that have not seen a GP previously for an AAD (Table 4). This increase corresponds with trends in alcohol-related hospitalizations that grew 15% from 378 persons per 100,000 to 437 persons per 100,000 in 2011 [32]. In this study, the largest increases in GP utilization from 2001–2011 were for alcohol abuse, alcoholic liver cirrhosis, and alcoholic fatty liver. The increasing number of persons presenting with alcohol abuse from 2001 to 2011 corresponds with trends in hospital discharges for alcohol abuse that grew from 4.8 per 100,000 in 2002 to 10.4 per 100,000 in 2011 [32].

There were also significant increases in the number of alcoholic fatty liver cases treated by GPs in BC from 3.3 cases per 100,000 in 2001 to 14.5 cases per 100,000 in 2011. Alcoholic fatty liver effects upwards of 20% of persons with alcohol dependency, and the increase in alcoholic fatty liver cases could be related to the large proportion of the sample having alcohol dependency [34]. In the 10-year period, the number of persons presenting to GPs with liver cirrhosis increased from 1.6 persons per 100,000 in 2001 to 8.9 persons per 100,000 in 2011. The results of this study suggest that cases of alcohol-related liver disease have been rising in BC since 2001 for both less complicated (alcoholic fatty liver) and more severe conditions (alcoholic liver cirrhosis). At the same time, mortality for alcohol-related liver disease has rose from 173 persons in 2003 to 304 persons in 2011 [24].

### Time series trends: service frequency

Although there were significant increases in the number of unique persons being treated by GPs for AADs from 2001–2011, there was also a decrease in the frequency of these visits. The mean number of visits per case peaked in 2007/2008, and subsequently declined for most AADs from 2009–2011 (Table 6). The timing of increases in the number of visits from 2007–2008 corresponds to marked increases in alcohol consumption and hospital discharges for alcohol-related diseases (2007–2009) [14] [32].

The declining frequency of GP visits since 2008 suggests that although more persons have AADs they are going to their GP less frequently for treatment. For example, the frequency of visits for alcohol dependence and alcohol abuse declined significantly from 2008–2011 to an average of 2.6 visits per person for treatment of alcohol dependence which fall below existing guidelines for treatment of these disorders. Screening and brief interventions for alcohol dependence require multiple visits to GPs and routine (e.g. monthly) follow up is recommended for medication management, referrals, and monitoring of alcohol consumption patterns post-intervention [35]. The results of this study show that many persons with AADs may not be receiving adequate levels of support as measured by the declining average number of visits to GPs. This could be partially attributed to increased referrals to specialists or tertiary level care that were not measured in this study, or associated with challenges to obtaining adequate primary health care support for treatment of AADs such as local physician shortages or patient reluctance to address drinking behaviors.

### Limitations

There are several limitations to the results of this study. The data modeled is physician-billing records for visits to GPs by persons with AADs and could be inaccurately categorized by ICD-9 code by health professionals. Hospital separations or visits to specialists were not included in our dataset, limiting our ability to understand the total magnitude of health care utilization for AADs in BC. The data does not include physicians that are paid using alterative payment schemes, such as salaried or sessional providers.

## Conclusion

From 2001 to 2011, there were significant increases in the number of persons presenting with AADs in BC, while at the same time significant decreases in the average number of visits per person. Additional research is needed to understand trends in health care utilization in the context of increasing AAD cases to determine why there have been significant decreases in the frequency of GP visits, and whether the current intensity of primary health care services is meeting patient demands and service needs. During this period there were also several regulatory changes to the distribution and price of alcohol in BC. While it is beyond the scope of this study to measure the direct impact of these policy reforms to health care utilization in BC, our findings suggest that the number of persons with AADs increased in the same period that liquor distribution was further liberalized throughout the province. The results of this study demonstrate the need for additional evaluative research on the direct impact of changes to liquor policy on alcohol consumption, the incidence of AADs, and health care utilization in BC.
